# Early assessment and analysis of high-risk factors of neurodevelopmental impairment in neonates with congenital diaphragmatic hernia

**DOI:** 10.3389/fped.2025.1632735

**Published:** 2025-09-12

**Authors:** Boliang Bai, Wendong Liu, Ronghui Yu, Xueping Zhu, Wenqiang Sun, Lang Jiang, Xiaodong Wang, Guanghao Su

**Affiliations:** Department of Orthopedics, Affiliated Children's Hospital of Soochow University, Suzhou, Jiangsu, China

**Keywords:** congenital diaphragmatic hernia (CDH), neurodevelopmental impairment, combined assessment, rSO_2_, LHR

## Abstract

**Background:**

This study aimed to retrospectively analyze clinical data of neonates with congenital diaphragmatic hernia (CDH) to investigate risk factors for neurodevelopmental impairment and their prognosis, and to evaluate the predictive value of combined assessment using amplitude-integrated electroencephalography (aEEG), regional cerebral oxygen saturation (rSO₂), and Neonatal Behavioral Neurological Assessment (*N*BNA) for early intervention.

**Methods:**

A total of 83 neonates with CDH (36 in the neurodevelopmental impairment group and 47 in the control group) were included, all diagnosed by prenatal ultrasound and postnatal imaging, with exclusion of other congenital malformations, hemodynamic instability, and genetic disorders. Clinical data [e.g., lung-to-head ratio (LHR), postoperative pulmonary hypertension, surgical approach], neuromonitoring indices (aEEG, rSO₂), and neurodevelopmental assessments (NBNA, Gesell Developmental Schedules) were collected. Independent risk factors for neurodevelopmental impairment and the area under the ROC curve (AUC) of aEEG, rSO₂, NBNA, and their combined assessment were analyzed.

**Results:**

Severe pulmonary hypoplasia (LHR < 1.5; OR = 6.20, 95% CI: 2.15–17.80, *P* = 0.005), postoperative persistent pulmonary hypertension (PPHN; OR = 2.80, 95% CI: 1.09–13.60, *P* = 0.027), and open surgery (vs. minimally invasive repair; OR = 2.80, 95% CI: 0.82–9.58, *P* = 0.056) were identified as independent risk factors for neurodevelopmental impairment in CDH neonates. aEEG scores and rSO₂ values in the neurodevelopmental impairment group were significantly lower than those in the control group at both 14 and 28 days (*P* < 0.001). The combined assessment of aEEG, rSO₂, and NBNA showed the highest AUC (0.960), with 83.0% sensitivity and 98% specificity.

**Conclusion:**

LHR < 1.5, PPHN, and open surgery are independent predictors of neurodevelopmental impairment in CDH neonates. The combined use of aEEG, rSO₂, and NBNA significantly improves the efficiency of early neurodevelopmental impairment identification (AUC = 0.960), outperforming single indicators. Clinicians should prioritize monitoring pulmonary hypoplasia and perinatal complications while adopting multimodal neuromonitoring to optimize early intervention strategies.

## 1 Introduction

Congenital diaphragmatic hernia (CDH), a life-threatening congenital anomaly with an estimated incidence of 1:3,000–5,000 live births (1). While advancements in perinatal care strategies—including optimized ventilatory management, targeted pharmacotherapy (e.g., milrinone and sildenafil), and judicious application of extracorporeal membrane oxygenation (ECMO)—have elevated survival rates to 70%–80% in tertiary centers (2), survivors face substantial neurodevelopmental morbidity. Prospective cohort studies reveal that 40%–60% of CDH survivors exhibit structural/functional neurological abnormalities (3–5), ranging from mild neurocognitive deficits to severe cerebral palsy, primarily attributable to prolonged cerebral hypoxia during critical care interventions (6).

Despite consensus on the multifactorial etiology of CDH-associated neurodevelopmental impairment—encompassing prenatal hypoxemia, postnatal hemodynamic instability, and treatment-related oxidative stress (7)—current predictive models demonstrate limited discriminative capacity (AUC = 0.62–0.71) (8). This diagnostic uncertainty stems from three key limitations: 1. overreliance on isolated neuroimaging findings with poor temporal resolution; 2. inadequate integration of multimodal neuromonitoring parameters; 3. paucity of longitudinal neurodevelopmental outcome data.

This study aims to retrospectively analyze the data of children with CDH during their hospitalization. It focuses on studying the high-risk factors for neurodevelopmental impairment and the prognosis of CDH children. Additionally, by examining the data of amplitude-integrated electroencephalogram (aEEG), regional cerebral oxygen saturation (rSO_2_), and Neonatal Behavioral Neurological Assessment (NBNA) during hospitalization, this study intends to explore whether multiple examination methods can improve the diagnostic probability of neurodevelopmental impairment in CDH children. The ultimate goal is to provide a reference for the early assessment and early intervention of neurodevelopmental impairment in CDH children.

## 2 Methods and analysis

### 2.1 General data

A total of 83 full-term neonates with CDH who were admitted to the Affiliated Children's Hospital of Soochow University and received surgical treatment from October 2019 to February 2024 were selected as the research subjects. All the infants underwent exutero intrapartum treatment (EXIT) at full term. This study has been approved by the Ethics Committee of the Affiliated Children's Hospital of Soochow University [Approval No.: 2025CS043].

#### 2.1.1 Inclusion criteria

1. Newborns diagnosed with CDH by prenatal ultrasound and re-diagnosed by x-ray and bedside ultrasound after birth;
2. Those with relatively stable basic conditions such as the circulatory system and pulmonary artery pressure and who can tolerate the surgery;
3. Those excluded from other congenital developmental malformations by bedside ultrasound examination;
4. Those for whom the EXIT technique was implemented by the same group of medical staff during childbirth, it should be noted that the application of EXIT procedures in our hospital is limited to establishing an airway before umbilical cord clamping, and does not include intrapartum ECMO catheterization;. A quality control team composed of experts from the Department of Obstetrics, Department of Pediatric Surgery and Department of Neonatology was established to ensure the standard implementation of the procedures;
5. According to the diagnostic criteria of brain damage syndrome (BDS) (9): Infants with high-risk medical history and meeting one of the following conditions: more than 3 items positive in the 36 items of the neurological examination from 1–12 months old, or epilepsy, inability of eyes to follow the light, and definite presence of one of the three items such as no directional response to the rattling sound; the total developmental quotient ≤ 85% or a single item ≤ 70%; one item positive in the 36 items plus the total developmental quotient ≤ 90% or a single item ≤ 80%; NBNA ≤ 35 points 28 days after birth (Supplementary Material S1). Infants meeting the above diagnostic criteria were included in the neurodevelopmental impairment group, and infants with high-risk medical history but not meeting any of the above conditions for the neurodevelopmental impairment group were included in the control group.

#### 2.1.2 Exclusion criteria

1. Other types of diaphragmatic hernia, such as hiatal hernia;
2. Hemodynamic instability after birth,: 1. manifested as continuous oxygen saturation < 85% when the inspired oxygen concentratio*n* > 50%; 2. mean arterial pressure < 30 mmHg accompanied by pale skin, cold skin, capillary refill time > 3 s, urine output < 1 ml/kg/h, lactate > 3 mmol/L; 3. pulmonary hypertension;
3. Genetic or chromosomal abnormalities related to neurodevelopmental delay;
4. Congenital abnormalities requiring other major surgeries;
5. Infants who died.

#### 2.1.3 Data collection

Including gender, gestational age, birth weight, Apgar score after birth, LHR, defect diameter (≥3 or not), duration of mechanical ventilation, and whether vasoactive drugs were used; presence or absence of intrauterine distress, maternal pregnancy complications (gestational hypertension, gestational diabetes mellitus, placental abruption, intrahepatic cholestasis of pregnancy); complications during the course of the disease (hypoglycemia, whether open surgery was performed, hypercapnia, anemia, pulmonary hemorrhage, postoperative pulmonary hypertension, sepsis, hyperbilirubinemia); rSO2 and aEEG scores at admission and 14 days after birth, NBNA 28 days after birth, and the results of Gesell Developmental Schedules for infants at 6 months and 1 year after birth.

### 2.2 Examination method

#### 2.2.1 NBNA

Before all newborns are discharged from the hospital or when they reach 28 days old, the Neonatal Behavioral Neurological Assessment (NBNA) scale (10) should be used to evaluate their neurodevelopment, including: Behavioral state regulation, Passive muscle tone, Active movement coordination, Primitive Reflexes, and General Neurological Status (Supplementary Table S3). The total score of the scale is 40 points, and a higher score indicates better nervous system development. This scale has good reliability and validity. In terms of reliability, it has a high test-retest reliability. The correlation coefficient of the scores for the same group of newborns in a short period of time is above 0.85, and the inter-rater reliability is around 0.80. In terms of validity, it can distinguish the level of development, and those with lower scores have a higher risk of abnormal nervous system development in the later stage.

#### 2.2.2 aEEG

The aEEG of newborns is recorded using the Lifelines video electroencephalogram system (USA). Before the tracing, the scalp should be cleaned first. According to the international 10–20 standard lead method, recording electrodes are respectively placed on the bilateral frontal lobes, central regions and temporal lobes of the infants. After disinfecting the placement sites, degreasing is carried out, and the conductive paste is injected into the electrodes, which are then fixed with a neonatal cap. For all infants in the neurodevelopmental impairment group, the recording is completed during their hospitalization. The monitoring time is 12 h each time. The first monitoring is carried out 48 h after birth, and then further monitoring is conducted on the 14th day and the 28th day after birth respectively. According to the improved aEEG scoring criteria (Supplementary Table S1) (11), the aEEG score is based on a total score of 12 points, including 5 points for Continuity, 3 points for the Sleep-Wake Cycling (SWC), and 4 points for Epileptiform Discharges. The lower the score, the more severe the neurodevelopmental impairment.

#### 2.2.3 rSO_2_

The brain and regional tissue oxygen saturation monitor (Jiangxi Yilude Medical Technology Co., Ltd., model/specification 01-06-3,000) is applied to conduct near-infrared spectroscopy (NIRS) monitoring of rSO₂ for newborns while performing aEEG monitoring. The probe is smoothly placed on the forehead (above the eyebrow ridge) (12) and fixed with medical adhesive tape. The probe at the monitoring site is shielded from light. After the probe signal is ≥2 grids and the graph is stable, the monitoring is carried out for 12 h and the average value is taken(Consistent with the aEEG monitoring period).

#### 2.2.4 Gesell

The Gesell Developmental Schedules are used to evaluate the intelligence, motor skills and behaviors of the children. It includes five functional areas: adaptability, gross motor skills, fine motor skills, language and social interaction. The Developmental Quotients (DQ) for each item = Developmental Age/Actual Age × 100. A DQ score of >85 points in each of the five functional areas is considered normal; a score between 75 and 85 points is regarded as marginal; and a score of<75 points indicates nervous system abnormalities. A DQ score of >85 points indicates a good prognosis, while a DQ score of <85 points or death indicates a poor prognosis.

### 2.3 Statistical analysis

This study used SPSS 26.0 for data analysis. For measurement data, the Shapiro–Wilk test was conducted to assess normality. Data conforming to a normal distribution were presented as mean ± standard deviation ($\bar{x}\pm s$), and inter-group comparisons were performed using the independent samples *t*-test. Conversely, non-normally distributed data were expressed as median (interquartile range) [M(P25, P75)], with Mann–Whitney *U* tests used for between-group comparisons. Categorical data were reported as frequencies and percentages [*n*(%)], and inter-group comparisons were carried out using the chi-square test; when the expected cell frequencies were less than 5, Fisher's exact test was applied.

For repeated-measurement data (such as aEEG scores and rSO₂ values) collected from the same group of newborns at 48 h, 14 days, and 28 days after birth, within-group comparisons were performed using repeated-measures analysis of variance (ANOVA). Mauchly's test of sphericity was conducted; if the sphericity assumption was violated, the Greenhouse-Geisser correction was applied. Pairwise comparisons among different time points within the group were adjusted using the Bonferroni method.

Receiver operating characteristic (ROC) curve analysis was utilized to evaluate the diagnostic efficacy of indicators, including aEEG scores and rSO₂ values, for neonatal neurodevelopmental impairment. The area under the curve (AUC) and its 95% confidence interval (CI) were calculated. The optimal cut-off value was determined based on the Youden index, and corresponding sensitivity, specificity, positive predictive value, and negative predictive value were computed. Delong's test was employed to compare the differences in ROC curves between various indicators.

Pearson correlation analysis was used to explore the relationships between variables, and a logistic regression model was applied for multivariate analysis to identify independent risk factors influencing neonatal prognosis. All statistical tests were two-tailed, and a significance level of *P* < 0.05 was set to determine statistical significance.

## 3 Results

In the initial stage of the study, a total of 88 infants were initially considered for inclusion. Among them, 5 infants were unable to be included in the study due to reasons such as hemodynamic instability or death. Finally, 36 infants were included in the neurodevelopmental impairment group, including 19 males and 17 females. The birth weight was (3,146.88 ± 432.06) g, and the gestational age was (270.30 ± 6.38) days. In the control group, 47 infants were included, with 23 males and 24 females. The birth weight was (3,180.02 ± 444.93.75) g, and the gestational age was (270.63 ± 6.19) days.

### 3.1 Comparative analysis of clinical characteristics between neurodevelopmental impairment group and control group

The study cohort consisted of 83 neonates. Demographic data analysis revealed no statistically significant intergroup differences in gender distribution, postnatal age at admission, gestational age at birth, birth weight, duration of mechanical ventilation, or the use of vasoactive agents (all *P* > 0.05). Moreover, defect diameter ≥ 3, perinatal maternal comorbidities, occurrences of intrauterine distress, and 1-minute Apgar scores were comparable across groups (*P* > 0.05). Similarly, no significant disparities were observed between the two groups regarding hypoglycemia, hypercapnia, anemia, pulmonary hemorrhage, hyperbilirubinemia, or sepsis (*P* > 0.05).

Notably, striking differences emerged in several key parameters between the two groups. In the neurodevelopmental impairment group, the proportion of neonates who underwent open surgery was 25%, while in the control group, it was 8.5%, with a statistically significant difference (*P* < 0.05). The prevalence of concurrent postoperative pulmonary hypertension in the neurodevelopmental impairment group was 27.8%, compared with 8.5% in the control group, and the difference was statistically significant (*P* < 0.05). Additionally, the incidence of pulmonary hemorrhage in the neurodevelopmental impairment group was 27.8%, vs. 10.6% in the control group (*P* < 0.05); the lung-to-head ratio (LHR) in the neurodevelopmental impairment group was 1.4 (1.4, 1.5), while that in the control group was 1.5 (1.5, 1.5), with all these differences being statistically significant (*P* < 0.05). These findings suggest that these factors may serve as potential risk factors for neurodevelopmental impairment in neonates with CDH (Table 1).

**Table 1:** Comparison of information between children in the neurodevelopmental impairment group and the control group.

Item		Neurodevelopmental impairment group	Control group	*χ²/t(Z)*	*P*
Gender (*n*, %)	Male	19 (45.2)	23 (54.7)	0.12^a^	0.729
	Female	17 (41.4)	24 (58.5)		
Gestational age (d, $\bar{x}\pm s$)		270.30 ± 6.38	270.63 ± 6.19	−0.239^b^	0.811
Birth weight (g, $\bar{x}\pm s$)		3,146.88 ± 432.06	3,180.02 ± 444.93.75	−0.340^b^	0.734
LHR [M(P_25_,P_75_)]		1.4 (1.4,1.5)	1.5 (1.5,1.5)	−3.271^c^	0.001
Defect diameter ≥ 3cm (*n*, %)		17 (41.4)	15 (31.9)	2.66^a^	0.103
Duration of mechanical ventilation (h, $\bar{x}\pm s$)		204.61 ± 21.339	206.74 ± 20.690	−0.459^b^	0.647
The use of vasoactive agents (*n*, %)		35 (97.2)	43 (93.6)	0.578^a^	0.447
Gestational hypertension (*n*, %)		17 (47.2)	14 (29.8)	2.648^a^	0.104
Diabetes mellitus (*n*, %)		20 (55.6)	30 (63.8)	0.583^a^	0.445
Placental abruption (*n*, %)		3 (8.3)	4 (8.5)	0.001^a^	0.977
Intrauterine distress (*n*, %)		5 (13.9)	9 (19.1)	0.402^a^	0.526
Cholestasis (*n*, %)		2 (5.6)	3 (6.4)	0.025^a^	0.875
1 min Apgar scores[M(P_25_,P_75_)]		8 (8,9)	9 (8,9)	−0.953^c^	0.341
Hypoglycemia (*n*, %)		10 (28.6)	13 (27.7)	0.008^a^	0.928
Open surgical repair (*n*, %)		9 (25)	4 (8.5)	4.196^a^	0.041
Hypercapnia (*n*, %)		7 (19.4)	13 (27.7)	0.752^a^	0.386
Anemia (*n*, %)		11 (30.6)	16 (34)	0.113^a^	0.737
Pulmonary hemorrhage (*n*, %)		10 (27.8)	5 (10.6)	4.045^a^	0.044
Hyperbilirubinemia (*n*, %)		28 (77.8)	40 (85.1)	0.739^a^	0.390
Postoperative PPHN (*n*, %)		10 (27.8)	4 (8.5)	5.397^a^	0.020
Sepsis (*n*, %)		4(11.1)	4(8.5)	0.158^a^	0.691

^a^ Represents the χ^2^ value.

^b^ Represents the *t* value, and

^c^ Represents the *Z* value.

Neurodevelopmental outcomes assessed by NBNA at 28 days postnatal demonstrated significant delays in multiple neurodevelopmental domains within the neurodevelopmental impairment group [Total score: 35(33, 37) vs. 38(37, 39), *P* < 0.001]. Domain-specific analysis revealed impaired performance in (Table 2): Behavioral state regulation [11(10, 11) vs. 12(12, 12), *P* < 0.001],Passive muscle tone [7(7, 7) vs. 7(7, 7), *P* = 0.004], Active movement coordination [7(5, 7) vs. 7(7, 8), *P* = 0.015].

**Table 2:** Comparison of NBNA scores between children in the neurodevelopmental impairment group and the control group [*M*(*P*_25_,*P*_75_)].

Grouping	Behavioral state regulation	Passive muscle tone	Active movement coordination	Primitive Reflexes	General Neurological Status	Total score
Neurodevelopmental impairment group (*n* = 36)	11 (10,11)	7 (7,7)	7 (5,7)	6 (5,6)	5 (5,6)	35 (33,37)
Control group (*n* = 47)	12 (12,12)	7 (7,7)	7 (7,8)	6 (5,6)	6 (5,6)	38 (37,39)
*Z*	−7.622	−2.895	−2.428	−0.195	−0.971	−6.041
*P*	<0.001	0.004	0.015	0.846	0.332	<0.001

### 3.2 Identification of independent risk factors for neurodevelopmental impairment *via* multivariable logistic regression

A comprehensive multivariable logistic regression model was constructed to evaluate 18 clinically pertinent variables, encompassing maternal-fetal comorbidities (gestational hypertension, diabetes mellitus, cholestasis), perinatal complications (placental abruption, intrauterine distress), and postnatal therapeutic interventions (vasoactive agent utilization, open surgical repair). Notably, all candidate predictors were retained in the final model via forced-entry method, irrespective of their univariate significance, to account for potential clinical interactions and confounding effects. Severe pulmonary hypoplasia (LHR < 1.5): OR: 6.200 (95% CI: 2.150–17.800, *P* = 0.005),Postoperative persistent pulmonary hypertension (PPHN): OR: 3.850 (95% CI: 1.090–13.600, *P* = 0.027), Open surgical approach vs. minimally invasive repair: OR: 2.800 (95% CI: 0.820–9.589, *P* = 0.056).Since the default value of 0.1 has been excluded, it can still be included as a high-risk factor, and further verification is required in subsequent steps (Table 3).

**Table 3:** Logistic regression analysis of high-risk factors for neurodevelopmental impairment in children with CDH.

Risk factors	β	*S* $\bar{x}$	*Wald_X_^2^*	*P*	*OR*	95% *Cl*
Severe pulmonary hypoplasia (LHR < 1.5)	1.820	0.650	7.830	0.005	6.200	2.150–17.800
Postoperative PPHN	1.350	0.610	4.870	0.027	3.850	1.090–13.600
Open surgical repair	1.030	0.540	3.650	0.056	2.800	0.820–9.589
Defect diameter ≥ 3 cm	0.760	0.510	2.210	0.137	2.410	0.790–5.810
Gestational hypertension						
Diabetes mellitus	−0.293	0.523	0.314	0.575	0.746	0.267–2.079
Cholestasis	−0.900	1.302	0.478	0.489	0.470	0.032–5.214
Placental abruption	0.215	0.854	0.064	0.801	1.240	0.233–6.607
Intrauterine distress	−0.294	0.706	0.173	0.677	0.745	0.0187–2.973
The use of vasoactive agents	0.320	1.293	0.061	0.804	1.377	0.109–17.371
Gestational age	−0.007	0.036	0.032	0.857	0.994	0.925–1.067
Birth weight	0.001	0.001	0.167	0.682	1.000	0.999–1.001
Duration of mechanical ventilation	−0.005	0.011	0.234	0.629	0.995	0.974–1.016
1 min Apgar scores	−0.168	0.310	0.295	0.587	0.845	0.460–1.553
Sepsis	−0.324	1.016	0.102	0.750	0.723	0.099–5.299
Hyperbilirubinemia	−0.600	0.685	0.767	0.381	0.549	0.143–2.102
Pulmonary hemorrhage	1.185	0.711	2.778	0.096	3.271	0.812–13.184
Anemia	−0.101	0.482	0.044	0.834	0.904	0.361–1.843
Hypoglycemia	−0.444	0.538	0.679	0.410	0.642	0.223–2.326

### 3.3 Comparative analysis of neuromonitoring parameters

The evolution of neural electrical activity measured by aEEG (Table 4): Longitudinal assessment,at admission, there was no difference in the aEEG scores between the two groups (Z = −0.922, *P* > 0.05), suggesting that the initial levels of neural electrical activity were comparable. At 14 days, the scores in the neurodevelopmental impairment group decreased significantly (median 11 vs. 12, Z = −4.977, *P* < 0.001), indicating the suppression of neural electrical activity. At 28 days, the scores in the neurodevelopmental impairment group were still lower than those in the control group (median 12 vs. 12, Z = −3.824, *P* < 0.001), reflecting persistent brain function impairment. By performing the rank sum test for the horizontal comparison within the group, it was found that the scores of the children in the neurodevelopmental impairment group decreased significantly (median 12 vs. 11 vs. 12, *η*² = 16.563, *P* < 0.001), further indicating the progressive brain function impairment in the children of the neurodevelopmental impairment group.

**Table 4:** Comparison of aEEG and rSO_2_ monitoring results of the two groups of children.

Grouping	aEEG [M (*P_25_, P_75_*)]			$\mathrm{rS}O_{2}\;(\bar{x}\pm s)$		
	Admission	14 d	28 d	Admission	14 d	28 d
Neurodevelopmental impairment group	12 (12,12)^a^	11 (10,12)^a^	12 (11,12)^a^	74.1 ± 5.24^b^	71.3 ± 4.75^b^	72.0 ± 3.58^b^
Control group	12 (12,12)	12 (12,12)	12 (12,12)	75.7 ± 5.59	75.7 ± 5.60	75.1 ± 4.21
Z/t	−0.922	−4.977	−3.824	−1.332	−3.779	−3.563
*P*	0.356	<0.001	<0.001	0.187	<0.001	<0.001

^a^ *η²* = 16.563 *P* *<* 0.001.

^b^ *F* = 5.263 *P* = 0.010.

Dynamic changes in rSO_2_ (Table 4): Longitudinal assessment, At admission, there was no difference in rSO_2_ between the two groups (Z = −0.922, *P* = 0.356), indicating that the initial levels of electroencephalogram activity were comparable. At 14 days, the rSO_2_ in the neurodevelopmental impairment group decreased significantly (71.3 ± 4.75 vs. 75.7 ± 5.60, t = −3.779, *P* < 0.001), suggesting an imbalance between oxygen supply and demand. At 28 days, there was still an oxygenation disorder in the neurodevelopmental impairment group (72.0 ± 3.58 vs. 75.1 ± 4.21, t = −3.563, *P* < 0.001). For the horizontal comparison within the group, analysis of variance for continuous measurements was used, and it was found that the rSO_2_ values of the children in the neurodevelopmental impairment group also showed significant differences (74.1 ± 5.24 vs. 71.3 ± 4.75 vs. 72.0 ± 3.58, F = 5.263, *P* < 0.05), further indicating that the autoregulation of cerebral blood flow in the children of the neurodevelopmental impairment group was impaired.

### 3.4 ROC curve analysis for neurodevelopmental impairment assessment

**Figure 1:**
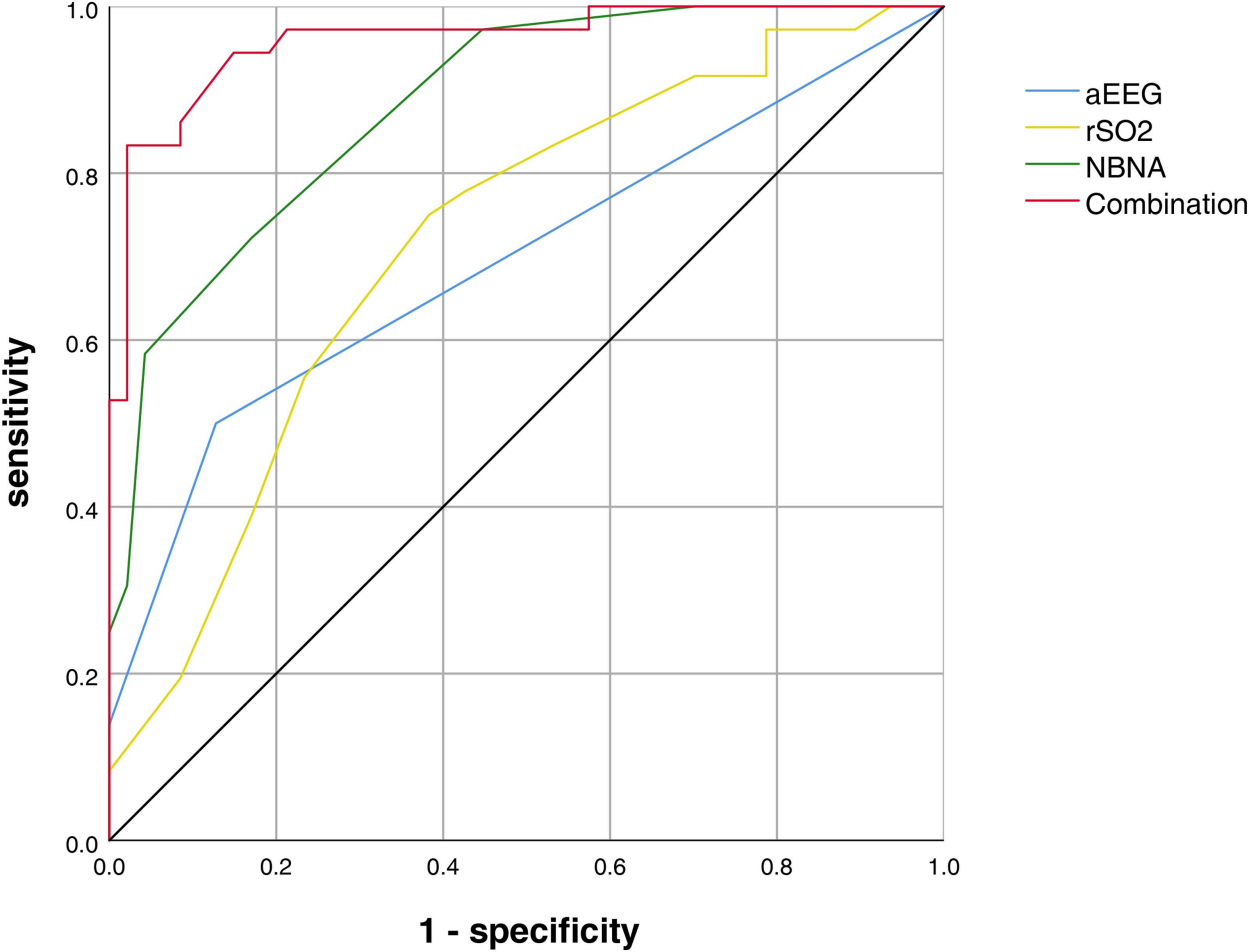
**ROC** curve analysis for neurodevelopmental impairment assessment.

As shown in the ROC curve analysis, the AUC for rSO_2_ at 28 days after birth, aEEG at 28 days after birth, NBNA score, and the combined assessment of neurodevelopmental impairment are 0.695 (sensitivity 50.0%, specificity 87%), 0.718 (sensitivity 75%, specificity 62%), 0.883 (sensitivity 72%, specificity 83%), and 0.960 (sensitivity 83.0%, specificity 98%) (Figure 1).

### 3.5 Longitudinal neurodevelopmental outcomes in CDH infants with neurodevelopmental impairment

The scores of the Gesell Developmental Schedules of 36 children in the neurodevelopmental impairment group at the age of 1 were statistically analyzed. One child died due to the recurrence of diaphragmatic hernia within one year of age, and two children were lost to follow-up within one year. The remaining 33 children were followed up for at least one year, with a follow-up rate of 91.6%. At 6 months of age, 17 children (accounting for 51.5%) had a Developmental Quotient (DQ) of >85 points in all five functional areas. At 1 year of age, 24 children (accounting for 72.7%) had a DQ of >85 points in all five functional areas.

A total of 42 children in the control group were included in the statistical analysis of the Gesell Developmental Schedules scores within 1 year of age. During the follow-up period, 1 child was lost to follow-up due to moving to another city within one year, and there were no deaths. The remaining 41 children completed at least 1 year of follow-up, with a follow-up rate of 97.6%. At 6 months of age, 30 children (accounting for 71.4%) had a Developmental Quotient (DQ) of >85 points in all five functional areas; at 1 year of age, 98 children (accounting for 90.4%) had a DQ of >85 points in all five functional areas. At 6 months of age, the proportion of children in the neurodevelopmental impairment group who achieved DQ > 85 points in all five functional areas was significantly lower than that in the control group; at 1 year of age, the compliance rate in the neurodevelopmental impairment group increased to 72.7%, but it was still lower than 90.4% in the control group, suggesting that the impact of neurodevelopmental impairment on the early neurodevelopment of children persists, and the overall developmental prognosis of the control group is better.

## 4 Discussion

In children with CDH, disordered airway branching during the fetal lung development leads to hypoplasia of both the ipsilateral and contralateral lungs, a reduction in the gas exchange surface, abnormal maturation of the cyst-alveoli, and abnormal muscularization of the distal arterioles. Moreover, the lung growth of CDH patients is impaired after birth. Even if surgery is performed in a timely manner, it cannot fully compensate for the initial pulmonary hypoplasia. These factors may collectively contribute to neurodevelopmental impairment in these children (13). In this study, in the neurodevelopmental impairment group of CDH patients, the scores of Behavioral state regulation were 11 (10, 11), the scores of Passive muscle tone were 7 (7, 7), and the scores of Active movement coordination 7 (5, 7), which were significantly lower than those in the control group. Additionally, during the follow-up from 6 months to 1 year of age, the scores on the Gesell Developmental Schedules showed developmental delay to varying degrees (51.5%, 72.7). The results of this study indicate that pulmonary hypoplasia has an impact on the brain development of children.

The results of this study are consistent with existing research. Among children with CDH, the most common neurological dysfunctions in adulthood include reduced muscle tone, hearing loss, impaired visuomotor function, oral-motor programming problems, behavioral attention disorders, inattentiveness, etc., with reduced muscle tone being more prevalent. Studies have shown that surviving children with CDH are at an increased risk of learning disabilities, and the neurodevelopmental outcomes of children who have undergone minimally invasive surgery are better than those who have received open surgery (14). The severity of the condition in children with CDH can predict their academic performance during the school-age period (15). The study by Takayasu (16) also showed that children who developed complications during treatment are more likely to have long-term complications. Friedman (17) reported that the duration of tracheal intubation is an independent predictive factor for the neurological prognosis of children with CDH at 1 year of age. Follow-up of children with CDH aged 3–7 years has found that their executive function scores and attention scores are lower than those of other populations (18). A meta-analysis has shown that among children with CDH, the retention rate of long-term motor deficits is 13%, the retention rate of abnormal cognitive function is 5%, the retention rate of abnormal hearing is 3%, and the incidence of neurodevelopmental impairment (15%) as well as the incidence of psychological problems (20%) are much higher than those in the general population (19, 20). However, the sample size of this study is limited, and the follow-up only extends to 1 year after birth. In the future, multi-center, large-sample, and long-term observational studies are needed to clarify the prognosis of children with CDH.

Currently, there are relatively few studies on the high-risk factors of neurodevelopmental impairment in children with CDH. This study has found that a LHR of less than 1.5, the presence of postoperative pulmonary hypertension, and the open surgical approach are high-risk factors for neurodevelopmental impairment in children with CDH. In the imaging evaluation of the prognosis of children with CDH, the LHR has received much attention (21). Measuring the LHR of children by ultrasound during pregnancy can predict the mortality rate of children with CDH during the perinatal period, with a sensitivity of 80%, a specificity of 73.5%, a positive predictive value of 47.1%, and a negative predictive value of 92.6% (22). Moreover, studies have shown that the survival rate of children with CDH is positively correlated with the LHR (r = 0.56, *P* < 0.001) (23). For example, among 380 children with CDH in Latin America, the LHR of the surviving children is significantly higher (56.5% vs. 34.9%; *P* < 0.01), which demonstrates the value of the LHR in predicting severe prognosis in children.Notably, the observed LHR should be interpreted in the context of gestational age-specific expected values (observed/expected LHR, o/e LHR), as validated by Huntley et al. (22). O/e LHR adjusts for fetal growth and gestational age, enhancing the accuracy of predicting pulmonary hypoplasia severity and neurodevelopmental outcomes.Future studies from our center will incorporate o/e LHR to further refine risk stratification for neurodevelopmental impairment. Pulmonary hypertension is one of the serious complications in children with CDH. The prognosis of CDH is related to the severity of initial pulmonary hypertension in newborns, and pulmonary hypertension usually decreases after the age of 5 (24). However, the incidence of pulmonary hypertension varies greatly among different centers (4.5%–38%). The more severe the pulmonary hypertension, the more the health-related quality of life declines (25). Approximately two-thirds of the surviving children with CDH have abnormal pulmonary function (20), because their impaired motor function and pulmonary function are related to the severity of postnatal pulmonary diseases. In this study, the incidence of PPHN in the neurodevelopmental impairment group (27.8%) was significantly higher than that in the control group (8.5%). Compared with previous studies, the incidence of PPHN in this study was slightly lower than that in the severe CDH cohort (35%-40%). However, multivariable regression confirmed that PPHN was an independent risk factor for neurodevelopmental impairment (OR = 3.850, 95% CI: 1.090–13.600, *P* = 0.027), which is consistent with international reports. This result suggests that PPHN is not merely a circulatory complication in children with CDH, but also an important early warning signal for neurodevelopmental impairment. In recent years, our center has been more inclined to endoscopic treatment when selecting the surgical approach for children. A meta-analysis shows that compared with the open surgery group, the mortality rate in the endoscopic treatment group is significantly lower (RR = 0.18, 95% CI: 0.09–0.38, *P* < 0.001), but the recurrence rate is significantly higher (RR = 3.10, 95% CI: 1.95–4.88, *P* < 0.001) (26). A retrospective analysis from 2011–2019 included 41 CDH patients who underwent surgery. The results showed that thoracoscopic repair can shorten the duration of mechanical ventilation and hospital stay and is conducive to the restoration of enteral nutrition, which is a safe and effective treatment method (27).This study found an association between open surgical approach and neurodevelopmental impairment (OR = 2.800, 95% CI: 0.820–9.589, *P* = 0.056), but this relationship requires careful interpretation within a clinical context. In our center's protocol, the use of open surgery was determined primarily by intraoperative findings indicating severe disease, such as large defect size (≥3 cm), significant pulmonary hypoplasia, or complex anatomical abnormalities. Thus, open surgery is more likely a marker of underlying disease severity rather than a direct cause of neurodevelopmental impairment. Further analysis revealed that infants undergoing open surgery frequently had lower LHR (indicating more severe pulmonary hypoplasia) and higher rates of postoperative pulmonary hypertension—factors independently associated with neurodevelopmental impairment. The observed association between open surgery and neurodevelopmental impairment may therefore be driven by these confounding high-risk factors.Future studies using propensity score matching or multicenter cohorts are needed to control for confounders such as defect size and pulmonary hypoplasia, to more accurately assess the potential impact of surgical approach on neurodevelopment. For high-risk infants requiring open surgery, enhancing perioperative brain protection (e.g., real-time rSO₂ monitoring, optimized cerebral perfusion) may be critical to improving outcomes.

There is no unified standard for neonatal neurodevelopmental impairment in the present research. Due to the underdeveloped behavioral functions of neonates, the early clinical manifestations of neurodevelopmental impairment are not typical, making the diagnosis quite difficult. Some studies have shown that 20% of neonates with neurodevelopmental impairment die, and 25% suffer from permanent neurological sequelae (28). Therefore, there is an urgent need in clinical practice to accurately identify the occurrence of neonatal neurodevelopmental impairment at an early stage, so as to guide clinicians to take active intervention measures, reduce or even block the apoptosis of nerve cells, and lower the mortality and disability rates of neonates (29). aEEG has been proven to be a valuable bedside monitoring tool for predicting the prognosis of neonatal neurodevelopmental impairment (30, 31). The aEEG score quantifies the monitored waveform according to its characteristics, and the modified aEEG score has been gradually formed during its application. The predictive value of neonatal aEEG for the neurological prognosis of neonates with hypoxic-ischemic encephalopathy and intraventricular hemorrhage has been reported (32, 33). However, aEEG has not been used as a routine monitoring method in clinical practice, and there are few reports on the prognostic value of aEEG changes for neonates during the perioperative period. This study takes neonatal diaphragmatic hernia as an example. The modified aEEG score of the first monitoring after birth in the neurodevelopmental impairment group is significantly lower than that in the control group. This decrease in score may be caused by the congenital malformation of the infant and the hypoxic state at birth. On the 14th day after birth, the modified aEEG score of the infants in the neurodevelopmental impairment group is significantly lower than before, and the score of the infants in the neurodevelopmental impairment group is also significantly lower than that of the neonates in the control group. With the improvement of the congenital malformation, nutrition, and other conditions of the infants after surgery, the modified aEEG score improves compared with before, but the overall score is still lower than that of the control group, indicating that the congenital pulmonary hypoplasia plays a crucial role in the neurodevelopment of neonates. NIRS is a non-invasive method that can be used for continuous bedside monitoring, has high safety, and can quantitatively reflect the local tissue blood supply. Currently, it has been applied in the fields of anesthesia, intensive care, and neonatology. In the field of neonatology, rSO_2_ detected by NIRS and its derived variables, fractional tissue oxygen extraction (FTOE) and splanchnic-to-cerebral oxygenation ratio (SCOR), the main parameters in international studies on local tissue oxygen metabolism. The main monitoring sites include the brain, kidneys, and abdomen (i.e., the intestine) to reflect the perfusion and oxygen metabolism of important organs in neonates (34). In a multi-center study in China in 2009, it was proposed that the measured value of cerebral oxygen saturation in normal full-term neonates is (62 ± 2)%, and a value lower than 58% indicates brain tissue hypoxia (27). There are few reports on the application of NIRS for the monitoring of neonates during the perioperative period in neonatal surgery. The monitoring results of this study show that in the short term after birth, the average value of the observation group is (73.8 ± 5.34)%, and the average value of the control group is (75.5 ± 5.84)%, which is consistent with the clinical manifestations and, combined with previous studies, is within the normal range. On the 14th day after birth, the rSO_2_ value of the neurodevelopmental impairment group decreases significantly. However, in the actual monitoring, the time when the decrease is most obvious is within 1–3 days after surgery. In this study, the modified aEEG score, rSO_2_, and Neonatal Behavioral Neurological Assessment (NBNA) are strongly correlated in the diagnosis of neurodevelopmental impairment in children with CDH. When used in combination, the area under the curve reaches 0.968, the sensitivity is 92.0%, and the specificity is 97.0%, showing a high diagnostic value. The combined diagnosis integrates the advantages of multiple detection methods. aEEG reflects the functional state of the brain from the level of electroencephalogram activity (30), rSO_2_ can monitor the oxygen supply of brain tissue in real time (35), and NBNA evaluates the neurodevelopment of neonates from the aspect of nerve behavior. The combination of the three can comprehensively judge neurodevelopmental impairment from multiple dimensions, improve the accuracy and reliability of the diagnosis, and reduce the occurrence of missed diagnoses and misdiagnoses. Especially for CDH infants with atypical clinical manifestations of neurodevelopmental impairment, the combined diagnosis can more sensitively capture abnormal signals, providing strong support for early intervention and contributing to the improvement of the prognosis of the infants.

At the same time, this study has certain limitations. Firstly, the sample size of the study is limited, and it may not fully represent the situation of all children with congenital diaphragmatic hernia, which may have a certain impact on the generalizability of the research results. rSO₂ was not dynamically monitored as a continuous variable, and the measurements taken at only two time points (14 days and 28 days) may not fully capture the association between its real-time fluctuations and neurodevelopmental impairment. Additionally, differences in the treatment stages of different children at the assessment time points may cause certain interference in the interpretation of the results. we did not systematically record the dosage, duration of use, and withdrawal time of sedative drugs, thus failing to evaluate their interference with aEEG scoring results. Neurological imaging examinations of the brain (such as cranial ultrasound and MRI) were not included in this study. Thus, we failed to evaluate the independent predictive value of imaging abnormalities for outcomes and their potential modifying effect on the association between included variables and outcomes. Secondly, the follow-up time is only up to 1 year after birth, and the assessment of the long-term prognosis of neurodevelopmental impairment in children with CDH is not comprehensive enough, making it difficult to determine the changing trends during the longer-term growth and development process. In addition, some assessment scales in the study, such as the NBNA and Gesell scores, may have a certain degree of subjectivity. Although the scales themselves have good reliability and validity, differences in the understanding and operation of the scoring criteria among different raters may still have a subtle impact on the results. The combined assessment also has certain drawbacks. In actual clinical applications, the combined assessment requires multiple examinations to be carried out simultaneously, and the operation is relatively cumbersome, which not only increases the workload of medical staff but also prolongs the examination time, potentially affecting the examination efficiency. Moreover, the cumulative cost of multiple examinations will increase the economic burden on the families of children. In addition, there is currently a lack of unified combined assessment criteria and operation specifications, and there may be differences in the implementation process among different hospitals and medical staff, which may affect the consistency and comparability of the combined assessment results. Future research needs to further expand the sample size, conduct multi-center and long-term follow-up observations, and adopt more objective and accurate assessment methods to explore in more depth the high-risk factors and prognosis of neurodevelopmental impairment in children with congenital diaphragmatic hernia, providing a more powerful basis for clinical treatment and intervention.

In conclusion, neurodevelopmental impairment in children with CDH may lead to poor prognosis, and the high-risk factors affecting neurodevelopmental impairment in children with CDH are relatively complex, involving congenital development, perinatal high-risk events, and the occurrence of complications during treatment. Therefore, the prevention of brain damage needs to be carried out throughout the entire period from pregnancy to the neonatal period. The medical team in neonatal surgery should be responsible for following up on children throughout the entire treatment cycle and intervening in a timely manner when abnormalities are detected. The combined application of aEEG scores, rSO_2_, and NBNA has a good application prospect in the assessment of neurodevelopmental impairment in children with CDH. However, during the promotion process, its advantages and disadvantages need to be fully considered, and the diagnostic process and standards need to be continuously optimized.

## Data Availability

The raw data supporting the conclusions of this article will be made available by the authors, without undue reservation.
