# Supplementary material for: Early assessment and analysis of high-risk factors of neurodevelopmental impairment in neonates with congenital diaphragmatic hernia
Source: Front Pediatr. 2025 Sep 12;13:1632735. doi: 10.3389/fped.2025.1632735 (PMC12463885; doi:10.3389/fped.2025.1632735)
Supplement: Supplementary file 1 [file Supplementaryfile1.docx]

| Supplementary Table 1. Modified aEEG scoring criteria | | | |
| --- | --- | --- | --- |
| Score | Continuity | Sleep-Wake Cycling (SWC) | Electrographic Seizures |
| 1 | Flat (isoelectric) | Absent | Status epilepticus: ≥30 continuous min |
| 2 | Continuous low voltage | Immature SWC | Recurrent: ≥2 episodes within 30 min |
| 3 | Burst-suppression | Mature SWC | Single episode |
| 4 | Discontinuous normal | - | None |
| 5 | Continuous normal | - | - |
