# Supplementary material for: Early assessment and analysis of high-risk factors of neurodevelopmental impairment in neonates with congenital diaphragmatic hernia
Source: Front Pediatr. 2025 Sep 12;13:1632735. doi: 10.3389/fped.2025.1632735 (PMC12463885; doi:10.3389/fped.2025.1632735)
Supplement: Supplementary file 2 [file Supplementaryfile2.docx]

According to the diagnostic criteria of brain damage syndrome (BDS): Infants with high-risk medical history and meeting one of the following conditions: more than 3 items positive in the 36 items of the neurological examination from 1 to 12 months old, or epilepsy, inability of eyes to follow the light, and definite presence of one of the three items such as no directional response to the rattling sound; the total developmental quotient ≤ 85% or a single item ≤ 70%; one item positive in the 36 items plus the total developmental quotient ≤ 90% or a single item ≤ 80%; NBNA ≤ 35 points 28 days after birth。

Grading: The grading criteria for Central Coordination Disorder can be referenced as follows:
① Very mild: 1-3 types of abnormalities in Vojta postural reflexes;
② Mild: 4-5 types of abnormalities in Vojta postural reflexes;
③ Moderate: 6-7 types of abnormalities in Vojta postural reflexes;
④ Severe: All 7 types of abnormalities in Vojta postural reflexes, accompanied by abnormal muscle tone.
